# Energetic Constraints Produce Self-sustained Oscillatory Dynamics in Neuronal Networks

**DOI:** 10.3389/fnins.2017.00080

**Published:** 2017-02-27

**Authors:** Javier Burroni, P. Taylor, Cassian Corey, Tengiz Vachnadze, Hava T. Siegelmann

**Affiliations:** ^1^Biologically Inspired Neural and Dynamical Systems Laboratory, College of Information and Computer Sciences, University of MassachusettsAmherst, MA, USA; ^2^Neuroscience and Behavior Program, University of MassachusettsAmherst, MA, USA

**Keywords:** neuronal metabolism, ATP, glia, epilepsy, Lotka-Volterra, ketosis, computational neuroscience, spiking neural networks

## Abstract

**Overview:** We model energy constraints in a network of spiking neurons, while exploring general questions of resource limitation on network function abstractly.

**Background:** Metabolic states like dietary ketosis or hypoglycemia have a large impact on brain function and disease outcomes. Glia provide metabolic support for neurons, among other functions. Yet, in computational models of glia-neuron cooperation, there have been no previous attempts to explore the effects of direct realistic energy costs on network activity in spiking neurons. Currently, biologically realistic spiking neural networks assume that membrane potential is the main driving factor for neural spiking, and do not take into consideration energetic costs.

**Methods:** We define local energy pools to constrain a neuron model, termed Spiking Neuron Energy Pool (SNEP), which explicitly incorporates energy limitations. Each neuron requires energy to spike, and resources in the pool regenerate over time. Our simulation displays an easy-to-use GUI, which can be run locally in a web browser, and is freely available.

**Results:** Energy dependence drastically changes behavior of these neural networks, causing emergent oscillations similar to those in networks of biological neurons. We analyze the system via Lotka-Volterra equations, producing several observations: (1) energy can drive self-sustained oscillations, (2) the energetic cost of spiking modulates the degree and type of oscillations, (3) harmonics emerge with frequencies determined by energy parameters, and (4) varying energetic costs have non-linear effects on energy consumption and firing rates.

**Conclusions:** Models of neuron function which attempt biological realism may benefit from including energy constraints. Further, we assert that observed oscillatory effects of energy limitations exist in networks of many kinds, and that these findings generalize to abstract graphs and technological applications.

## 1. Introduction

With health and basic biology in mind, many neurological problems are co-morbid with metabolic disorders, such as diabetes (Reske-Nielsen and Lundbæk, 1963; Dejgaard et al., 1991; Biessels et al., 1994; Duby et al., 2004; Mijnhout et al., 2006). Further, energetic metabolism can have a huge impact on brain function and neurological diseases (Gasior et al., 2006; Barañano and Hartman, 2008; Stafstrom and Rho, 2012). With biologically inspired technology as a goal, the brain requires a large proportion of the body's energy, and yet is highly efficient for its absolute power compared to modern supercomputers, with the brain consuming orders of magnitude less energy for more functional processing.

### 1.1. Biological energy in brains

Biological neurons are fundamentally cells first, and neurons second, with high energy requirements. A newborn human weighs around 3.5 kg with a brain about 400 g, which consumes about 120 kcal/day, a disproportionate 75% of total caloric intake (Holliday, 1971); during adulthood, an average human weighs around 70 kg, and the brain about 1, 400 g, which consumes about 415 kcal/day, a slightly less but still quite disproportionate 23% of total calories. In other words, the human brain is <2% of the body's volume but burns 23% of daily calories.

#### 1.1.1. Neuron energy budgets

With around 86, 000, 000, 000 neurons in the human brain (Herculano-Houzel, 2011a) each neuron consumes in the range of 4.8 × 10^−6^ cal/day. When considering experimental data in rodents, of the total energy consumption by gray matter (mostly neurons; at a rate of around 2.22 × 10^9^ ATP/neuron/s), spikes account for around 20% of the gray matter budget, with postsynaptic effects 50% (Howarth et al., 2012); notably this excludes housekeeping metabolic costs. Each neuron may fire around 350, 000 times/day at a rate ranging from [0.15 to 16 Hz], with means from [1.5 to 4 Hz] (Fanselow and Nicolelis, 1999; Schoenbaum et al., 1999). Each action potential may consume around 1.19 × 10^8^ ATP, and with a firing rate of around 4 Hz, spiking alone consumes roughly 4.75 × 10^8^ ATPs/neuron/s (Howarth et al., 2012). One (action potential/cortical neuron/s) raises oxygen consumption by (145 mL/100 g gray matter/h) (Attwell and Laughlin, 2001). Notably, neuronal anatomy and physiology are extremely variable within species (Markram et al., 2015) as are architectures and glial support across species (Herculano-Houzel, 2011a; Han et al., 2013), and as neuronal energy requirements also must be (Lennie, 2003). The animal and human numbers discussed here provide a theoretical context, and define rough ranges of operation for our upcoming model, as follows.

For example, out of activity-dependent energy consumption, most is consumed by the synapse itself (Rangaraju et al., 2014), where typical energy in a pool at synapse is around 10^6^ free ATP per nerve terminal. Further, despite this reserve, ATP production and transport responds quickly to synaptic activity, and disruption of ATP regeneration can impede activity rapidly, perhaps in part due to the dual role of ATP, of both signaling and energy supply (Rangaraju et al., 2014; Lindberg et al., 2015). In our upcoming model, the ratio of spike cost to available energy can be used to compute the rough number of spikes required to impede firing; these biological data suggest that in the model, pool depletion should be rapid in the absence of regeneration.

#### 1.1.2. Glial function: metabolism, energy, and more?

Glia are deeply intertwined with neurons and perform many functions, including providing metabolic support for neurons, controlling synapse formation, modifying blood flow, neuromodulation, and various other roles (Barres, 2008). They even have their own class of “gliotransmitters” which signal to neurons (Araque and Navarrete, 2010; Perea et al., 2014). Quite intriguingly, grafting human glial precursors into mice during development makes them learn faster and appear generally more intelligent, enhancing long-term potentiation (LTP) and performance on a variety of tasks; in a control condition grafting mouse cells similarly does not enhance learning (Han et al., 2013). Thus, it has been suggested that the human glia provided some information-processing improvement over rodent glia.

#### 1.1.3. Measuring brain resources in humans: PET, fMRI, CBF, BOLD

Metabolic effects can be measured in living humans via methods like positron emission tomography (PET) or functional magnetic resonance imaging (fMRI), producing indicators of glucose and ketone uptake, or blood oxygen level dependent response (BOLD) on cerebral blood flow (CBF). The relationship between neuronal activity and CBF is defined as neurovascular coupling, and this relationship is often considered to be non-linear (Pasley and Freeman, 2008). For example, a certain quantity of spikes or local field potentials elicited by sub-threshold activity or neuromodulation may result in a proportional quantity of increased blood flow (Raichle and Gusnard, 2002). Notably, the resultant increase in blood flow is greater than would be predicted by oxygen use alone (Leithner and Royl, 2014), which in light of this context, suggests the need for waste product removal or other factors. Spikes and sub-threshold responses produce different reactions in CBF, where with consistent activity in a region, local field potentials (LFPs) and blood flow remain high and well-coupled, with spikes tapering off, as if they were more metabolically expensive (Mathiesen et al., 1998; Logothetis et al., 2001; Logothetis and Wandell, 2004; Viswanathan and Freeman, 2007). Sub-threshold depolarizations and synaptic activity may also in part drive the CBF. At a whole brain level, measured via such means, basal metabolism is estimated to consume 30% of brain glucose, with spontaneous brain activity consuming around 70% of the brain's energy (Tomasi et al., 2013).

#### 1.1.4. Ketosis, metabolism, epilepsy, and neurological diseases

Metabolism may impact many neurological disorders. A ketogenic diet is hypothesized to be the oldest documented therapeutic diet, first noted as a treatment for seizures by Hippocrates. Currently, a highly reliable treatment for seizures, particularly the drug-resistant form, is a ketogenic diet (Yellen, 2008; Zupec-Kania and Spellman, 2008; Rho and Stafstrom, 2012; Danial et al., 2013; Korsholm and Law, 2013; Lutas and Yellen, 2013). When fasting, or consuming a diet very low in carbohydrate and high in fat, the body will produce ketone bodies which produce 2× ATP/molecule as glucose, as well as glucose, to fuel the brain; other organs and muscles temporarily use ketones but transition to full use of fatty acids leaving the brain as the primary consumer of ketone bodies in a long-term ketogenic diet. Note, dietary ketosis is distinctly different from diabetic ketoacidosis.

Intentional dietary ketosis may be the most globally impactful metabolic intervention for brain function known, having broad physiological impact on neurons, brains, and a surprising multitude of disease outcomes, including epilepsy, malignancy, trauma, stroke, neurodegeneration, Parkinson's, Alziemer's, ALS, Autism, depression, migraine, insomnia, aging, mitochondrial diseases, and more (Gasior et al., 2006; Barañano and Hartman, 2008; Stafstrom and Rho, 2012). Brain regions which consume the most energy (Sokoloff, 1991, 1996), fire the most rapidly, and have the highest incidence of seizure. Inhibitory neurons are about 10–15 times more connected than excitatory neurons, and they make many more local connections. Thus, with inhibitory transmission being proportionally more metabolically expensive than excitatory per neuron, if inhibitory neurons run out of energy differentially, it could relax suppression of excitatory firing.

### 1.2. Energy-related models in biological networks

Since there is virtually no literature computationally modeling the direct effect of energy on network activity with spiking neurons, we extend the background to other domains.

#### 1.2.1. Glia models: non-energetic

In addition to metabolic support, glia possibly play bulk neuromodulatory role in neuronal processing, and few studies have attempted to model this algorithmically, though without the primary goal of exploring energy requirements on spiking networks (Porto-Pazos et al., 2011; Allam et al., 2012; Alvarellos-González et al., 2012; Volman et al., 2012). These studies are interesting, in part because they improve neural network performance on AI-like tasks via incorporating signaling algorithms thought to be performed by glia, much as in murine experiments discussed above (Han et al., 2013).

#### 1.2.2. Neuron models: competitive, but non-energetic

It is possible to consider that artificial neural networks may have modeled something like resource constraint more abstractly; competition between neurons for long term potentiation and depression resources may recapitulate some features of energy restriction (Hunzinger et al., 2012). Further, via inhibition, other forms of competition may take place (Song et al., 2000; Atsumi, 2003; Behi et al., 2012; Ruan et al., 2012; Tal et al., 2014). As another example, energy can be considered in a non-analogous sense, wherein physics-inspired computation is defined in an orthogonal manner, such that “energy” is used for computation and/or memory (Bengio, 2015), which may occur (Lindberg et al., 2015), but is not a resource *per-se*. Though competition can impact network dynamics, it misses features of resource regeneration as well as energetic dynamics.

#### 1.2.3. Ecological resource models: predatory-prey dynamics

One might consider that resource models abstractly could speak to formalizing energetic dependence in neurons or brains. Ecology has modeled predator-prey dynamics, which may resemble resource constraints in biological networks broadly. In this type of model, a predator (e.g., wolves) and prey (e.g., rabbits) interact in a network of organisms distributed throughout the physical environment, with the prey serving as the resource, which limits the predator.

These systems are often modeled via Lotka-Volterra equations (Lotka, 1910; Goel et al., 1971). These are non-linear, differential equations: $\frac{dx}{dt}=\alpha x-\beta xy$ and $\frac{dy}{dt}=\delta xy-\gamma y$, where: *x* is number of prey; *y* is number of individual predators in a species; $\frac{dy}{dt}$ and $\frac{dx}{dt}$ are growth rates of two populations over time; *t* is time; and α, β, γ, δ are positive real parameters. This system will often produce a limit-cycle in population sizes between the predator and prey.

Tangentially, though not in study of resource constraints, neural network activity itself has been modeled using Lotka-Volterra methods (Noonburg, 1989) which themselves can describe many types of dynamical systems. Further, approximating neural network activity using these means may allow for more formal analysis of neural network activity (Moreau et al., 1999). Though these models describe natural oscillation in neuronal networks, none included energetic constraints.

### 1.3. Models of energy in technical, *in-silico*, hardware, economic, or abstract networks

Models of resource or energy usage and constraint in other networked systems may also provide insight into modeling neuroenergetics. Lotka-Volterra equations have also been used in economics for many years to model resource interactions between industries and sectors (Gandolfo, 2008), which may produce oscillatory interactions as well. Within graph theory, related questions include how to maximize graph connectivity with limited or unreliable functional links between nodes (Wan et al., 2008), a task which the brain may perform. There are practical applications in maintaining functional connectivity in networks, particularly in telecommunications (Shtessel et al., 2012) robustly, as brains do. Resource constraints are also applied via load-balancing and sharing protocols and algorithms to allocate network resources, power, CPU, disk drives, including shortest path bridging (Allan et al., 2010; Arpaci-Dusseau and Arpaci-Dusseau, 2015), some of which may show distant similarities to energetic constraints on maintaining brain functional connectivity (Tomasi et al., 2013).

### 1.4. Our contribution: models of resource constraint in spiking neurons

Though much is known about the impact of energy resources on mammalian brains, and there have been some attempts to model other functions of glia, there have been no studies directly modeling resource-dependence in computational neural networks. Despite the popular practice of modeling seizures in neural networks (Richardson, 2012), and the strong impact of ketone-bodies and energy metabolism on the treatment of seizure, there has been no attempt to include explicit energy in epilepsy spiking neural network models. Here we remedy the dearth of research into the effect of resource-dependence on biologically realistic neural networks.

### 1.5. Definitions of energy and resources

In biological systems, distinctions can be made between various resources: glucose, ketone bodies, ATP, glycogen, pyruvate, oxygen, lactate, neurotransmitter production, and various forms of waste-removal such as *CO*_2_ or free radicals. Ultimately, just as phenomenological neuron models can mimic neuronal behavior in a model-free descriptive sense, energy resources ultimately behave as a pool with delayed momentum both in refilling and regeneration. We chose an abstract level for our model such that it does not speak to the specific constituents of biological energy, but generalizes to explore this pool-like resource utilization by neurons and networks broadly, and resource-constraint abstractly.

## 2. Materials and methods

### 2.1. Base neuronal model with energy pool

We designed an energy pool for a biologically realistic neuron model, termed Spiking Neuron Energy Pool (SNEP). The well-studied phenomenological neuron model we used is in the family of Spike Response Models (SRM), which is a generalization of integrate-and-fire models. We added an energy pool which neurons utilize and deplete when spiking (Equations 1–3). Each neuron has one such corresponding energy resource pool. Energy pools can range from empty to full. Each spike requires energy and drains the resource pool in a pre-defined quantity, which is parametrically varied in our experiments. Resources in energy pools replenish, either at constant or varying rates. As typical, each neuron maintains a memory of its membrane potential, integrates new signals from synaptically weighted input spikes via changes in membrane potential, and spikes down one-directional axons when the membrane potential threshold is crossed (i.e., becomes positive enough in voltage), but with the caveat that firing is possible only if its energy pool contains more than a lower bound of sufficient resource. Therefore, every spike reduces energy in the pool, and the pool replenishes over time. When a firing event happens, action potentials are transmitted through the neuron's axons and synapses to its neighbors in a quantity proportional to the synaptic weight between each pair of neurons, increasing the neighbors' probability of firing. Synaptic weights do not change with experience; thus, there is no plasticity. If a firing neuron is an inhibitory neuron, the same mechanism applies, but the receiving neuron's potential moves away from the firing threshold (negative). Inhibitory neurons are 20% of the population, matching data in both rodents and primates (humans). Each spike takes time to reach its destination, and this time is a function of the distance between the source and target neurons. A small probability of sub-threshold firing is adjusted proportional to the membrane potential. For computational efficiency, a step-wise, rather than continuous, refractory effect limits neurons from firing shortly after they have just fired. In practice, within large ranges of these energy constraints, neurons will often be restricted from firing based on energy limitation, when they would have fired based on membrane potential alone. To conclude, typically a neuron model would fire singly due to its membrane potential, but in our model a second factor, energy, serves to regulate spiking.

(1) $$\begin{array}{r} \text{Change in membrane potential: }{\overset{\cdot }{V}}_{k}=-V/\tau_{\nu }+\underset{j}{\sum }w_{jk}\times I_{j} \end{array}$$

(2) $$\begin{array}{r} \begin{array}{c} \text{Change in energy pools}\\ \;(\text{replenishment}): \end{array}\;ė=\left\{ \begin{array}{cc} \tau_{e}\; & \text{if }e<e_{max}\\ 0\; & \text{if }e\geq e_{max} \end{array}\right. \end{array}$$

(3) $$\begin{array}{r} \begin{array}{c} \text{Spike conditions}:\text{if}:V>V_{\theta }\text{ and }e>r_{e}\text{ then}:V=V_{r},\\ \;e=e-r_{e},\text{ and initiate a }spike. \end{array} \end{array}$$

### 2.2. Definitions

- *V* is the present dynamic membrane potential (voltage) of a neuron, with *V*_*k*_ indexing the signal-integrating neuron.
- *I*_*j*_ is employed for the set of input signals from pre-synaptic neurons, indexed by the pre-synaptic neuron, *j*
- *w*_*jk*_ indexes the set of weights connecting input neurons, *j*, to the signal-integrating neuron, *k*.
- *V*_*r*_ is the negative resting membrane potential, to which the neuron returns after spiking.
- *V*_θ_ is the firing threshold for an action potential, which if *V* goes above, enables a spike.
- τ_*v*_ is a small positive constant, and specifies the rate of leak in membrane potential from *V*_*r*_ toward *V*_θ_; thus, a given neuron can eventually fire in the absence of inputs (similar to random biological firing).
- *e* is the current dynamic quantity of resources in an energy pool.
- *e*_*min*_ and *e*_*max*_ are the minimum and maximum functional limits for energy in the pool. When energy is less than *e*_*min*_ the cell fails to operate normally via integrating inputs (i.e., not reacting via membrane potential). When energy is at *e*_*max*_ replenishment ceases.
- *E* is the total amount of energy in all pools summed (remaining energy).
- *r*_*e*_ is the energetic cost of a single spike. A pool requires at least *r*_*e*_ for a neuron to spike, and *r*_*e*_ is subtracted from the pool after spiking. When energy in the pool is less than *r*_*e*_ the cell will not spike, even if its membrane potential is beyond the spiking threshold.
- τ_*e*_ is an assumed linear rate at which the energy pool is replenished with resources used by spiking neurons, specified in quantities of an arbitrary unit, γ.
- γ is defined as an arbitrary unit of energy, and can be conceived of as something akin to combined effect of ATP, glucose, ketone-bodies, oxygen, neurotransmitter production, and waste-removal. In other words, energy in this context represents resource abstractly.

### 2.3. Network structure

Though network structure and energy parameters are largely independent, structural network density could impact neuronal transmission. Macro-level connectome data are very well documented in human subjects (Nooner et al., 2012), and at a large scale, graph features are similar to those in rodent brains. At a micro-scale, neuronal density in primate (human) brains is much greater than in rodents (Herculano-Houzel, 2011a), which likely affects neuronal transmission. To study dynamical effects of energy dependence of neurons, we incorporate the SNEP's into a large neural network with structure and weight distribution from *in-vivo* biological macro-connectome data derived from rsfMRI and DTI in human participants (Nooner et al., 2012; Taylor et al., 2015), since these whole-brain data are well-validated.

Our neural network consists of 7, 500 explicit energy-dependent spiking neurons. Clustering was performed via spectral methods (Craddock et al., 2012), which have been previously demonstrated at a resolution of around 200 regions. To obtain around 40 neurons per cluster, sub-groups of neurons were clustered into 188 regions, and inside each region, neurons were connected with a probability that is inversely proportional to distance. Between regions, the probability of two neurons laying in different regions to be connected is proportional to the connectivity matrix used in Taylor et al. (2015). Weight distributions are initialized according to realistic distributions, and constants such as leak rate default to standard rates in the software simulation. In this model, axonal connections between neurons have directionality. The size of the network can be scaled up.

### 2.4. Simulation environment: BrainPower

**Figure 1:**
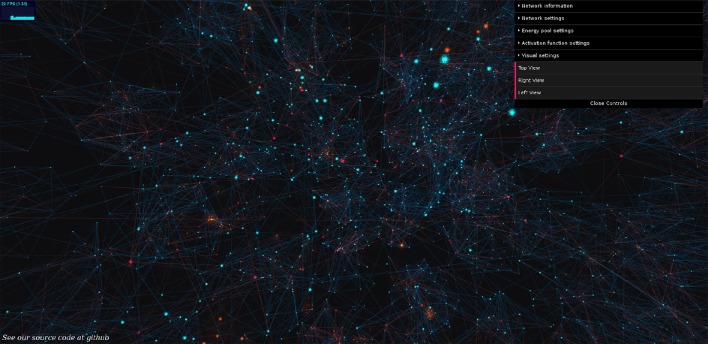
**BrainPower: our open simulation environment**. Neurons are orbs of light, axons are shafts of light, blue for excitatory, and red for inhibitory, and active signals (action potentials) move along axons as traveling diffuse golden clouds. We extend this 3D simulation and visualization in JavaScript using Python, and a fully functional version of our simulation can be used by anyone live at: https://binds.cs.umass.edu/BrainPower/. Neural network activity is visualized in real-time, and runs locally in most modern web-browsers. Drop-down control menus on the right top of the simulation allow a user-interactive way to set network parameters; each menu can be expanded to an extensive set of options.

The simulation, coined “BrainPower,” allows great flexibility in the choice of neuronal parameters and energy dynamics (Figure 1). Manipulable neuronal parameters include an extensive set of features (below). Manipulable energy parameters include regeneration rate, the energy requirement per spike, and the oscillatory type of energy regeneration as static or oscillating.

Users can explore the simulation freely, and it runs locally in the web browser. This provides two benefits, (1) the simulation can be widely shared without installation, and (2) the code is consistent across experiments and platforms since it is hosted on the remote server. The set of GUI-parameters is displayed at right in the interface, and each is commented internally. Data-logging is built in as an advanced feature with a Python back-end. A demo is available at: https://binds.cs.umass.edu/BrainPower/, and the open source code is freely available under the MIT license at: https://gitlab.com/BrainPower/Neural-Network/.

### 2.5. Experimental design

#### 2.5.1. Independent manipulated variables

Our approach is to systematically map the space of energy and neuronal parameters, covering biological ranges, and extending parameters widely enough to describe the effect of energy on network dynamics generally. For example, with *r*_*e*_ = 0γ the network will typically remain active for long periods of time, while with very high energy requirements for a spike, activity can dampen or die out, with a continuum of impact in between these ranges. We describe both likely biological ranges, and also those parameter extremes which may have technological relevance outside of biology or neuroscience.

We manipulate the energetic parameters, *r*_*e*_, τ_*e*_, and neuronal parameters, such as *V*_θ_, to explore the effect of energy on network dynamics. We vary *r*_*e*_ between *r*_*e*_ = 0γ and *r*_*e*_ = 0.4γ. For the first experiments, we set replenishment rate as a fixed value, with τ_*e*_ = 0.003γ/*ms* or τ_*e*_ = 0.006γ/*ms*. Each value of *r*_*e*_ is run as a separate simulation for 40,000 iterations, or 40 s where each iteration represents 1 ms.

#### 2.5.2. Dependent measured variables

With multiple 40 s experiments, we analyze the last 10 s of every simulation, discarding the first 30 s of data to ensure the convergence to a stationary process. A stationary process is defined as a process in which the joint distribution of the random variables at times *t*_1_ + τ, *t*_2_ + τ + …, *t*_*k*_ + τ does not depend on τ. Therefore: *F*_*X*_(*x*_*t*_1_ + τ_, *x*_*t*_2_+τ_, …, *x*_*t*_*k*_+τ_) = *F*_*X*_(*x*_*t*_1__, *x*_*t*_2__, …, *x*_*t*_*k*__). Once the simulation reaches a stationary process, it behaves similarly indefinitely. Thus, we can analyze its behavior using only a finite size sample. When no energy constraint is applied to the system, the stationary process is such that the level of activity of the network follows a white noise distribution, but with energy constraints it takes time to reach a stationary process.

We analyze time series variables of *total firing activity* and *available energy*. To measure total activity of the network, we record the number of spiking events at every time step, indexed via neuronal identity. Equivalently, to measure the available energy at every time step, we record energy in each pool in quantity of γ. This number represents the quantity of energy resources available for potential firing.

### 2.6. Simulation parameters

The following parameters are used for our simulations (further documented within the freely available software). The value for “*fireEnergy*” below represents *r*_*e*_, the variable we use for our analysis. The replenish energy τ_*e*_ can be made to oscillate between an upper and lower limit by modifying “*maxThreshold*” and “*minThreshold*” but we fix it to two levels, 0.003 and 0.006γ/*ms*, to have a constant rate of replenishment in these first studies.

**Energy pool settings:** amplitude: 0.01, fireEnergy: manipulated, frequency: 100, lastRegenUpdate: 0, maxEnergy: 1, maxThreshold: 0.003, minEnergy: 0, minThreshold: 0.003, regenSign: 1, regenerationTime: 1, replenishEnergy: 0.003.

**Network settings:** AxonDistance: 10, decayTime: 1, firing_threshold: 0.6, izhikevich: False, refactoryPeriod: 10, shouldLog: False, signal_weight: 0.4.

## 3. Results

### 3.1. Energy limitations introduce oscillatory dynamics

**Figure 2:**
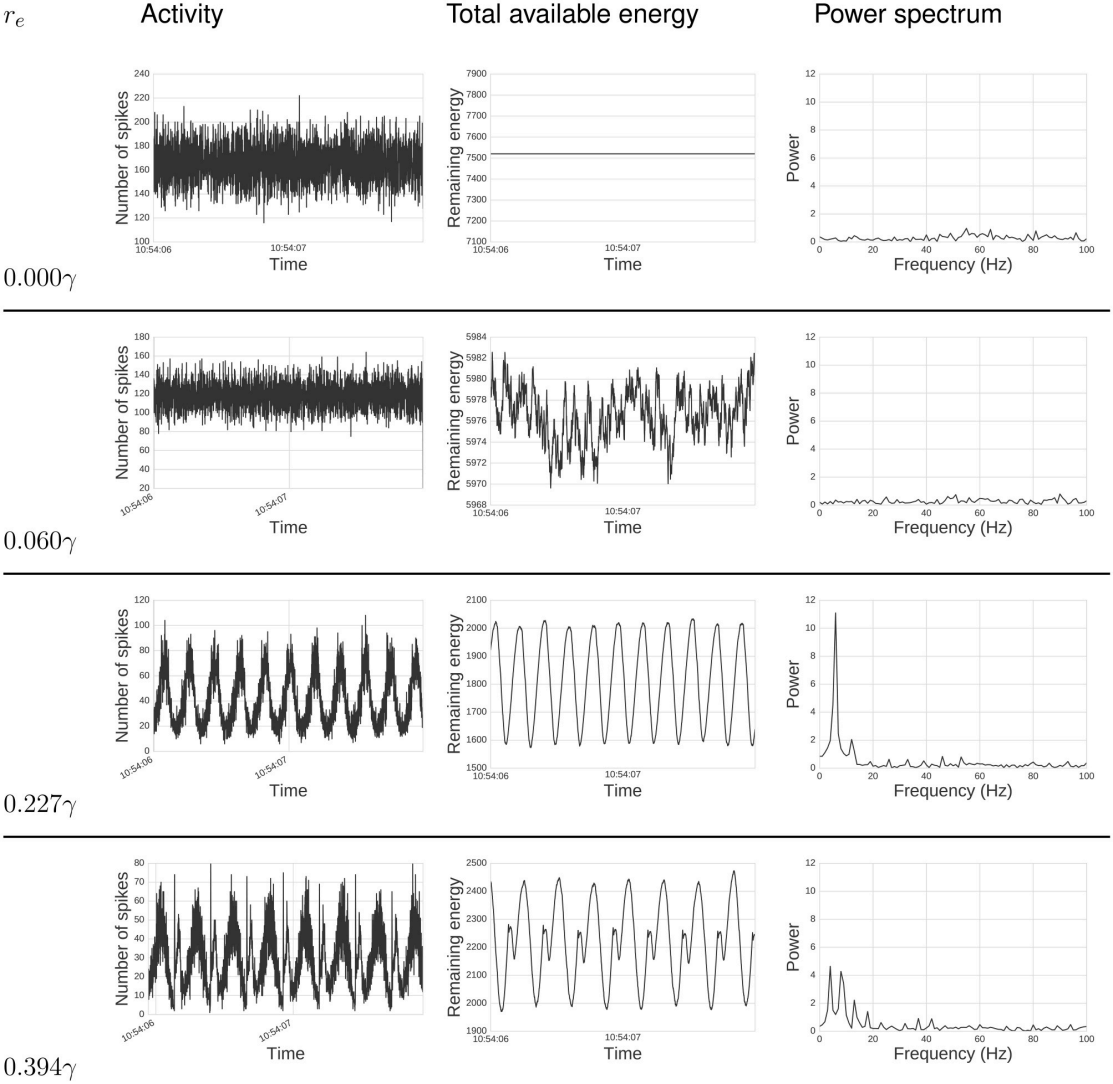
**Energy impacts activity**. Top text specifies: total activity **(left)**, total available energy **(center)**, and power spectrum **(right)** under different energy requirements, *r*_*e*_ values, for a period of 2s. From top to bottom, *r*_*e*_ goes from zero required energy *r*_*e*_ = 0γ to a relatively high requirement *r*_*e*_ = 0.394γ. When *r*_*e*_ = 0γ, the total activity behaves much like random noise, while the available energy remains constant, since there is no cost for spiking. As we increase *r*_*e*_, it can be seen that activity synchronizes, creating oscillatory behavior which intensifies with large *r*_*e*_. The power spectrum analysis also shows this synchronization. When *r*_*e*_ is zero or small, there is no single frequency displaying high power. As we increase *r*_*e*_, power gets concentrated in fewer frequencies, and these frequencies are related to *r*_*e*_, as discussed below.

**Figure 3:**
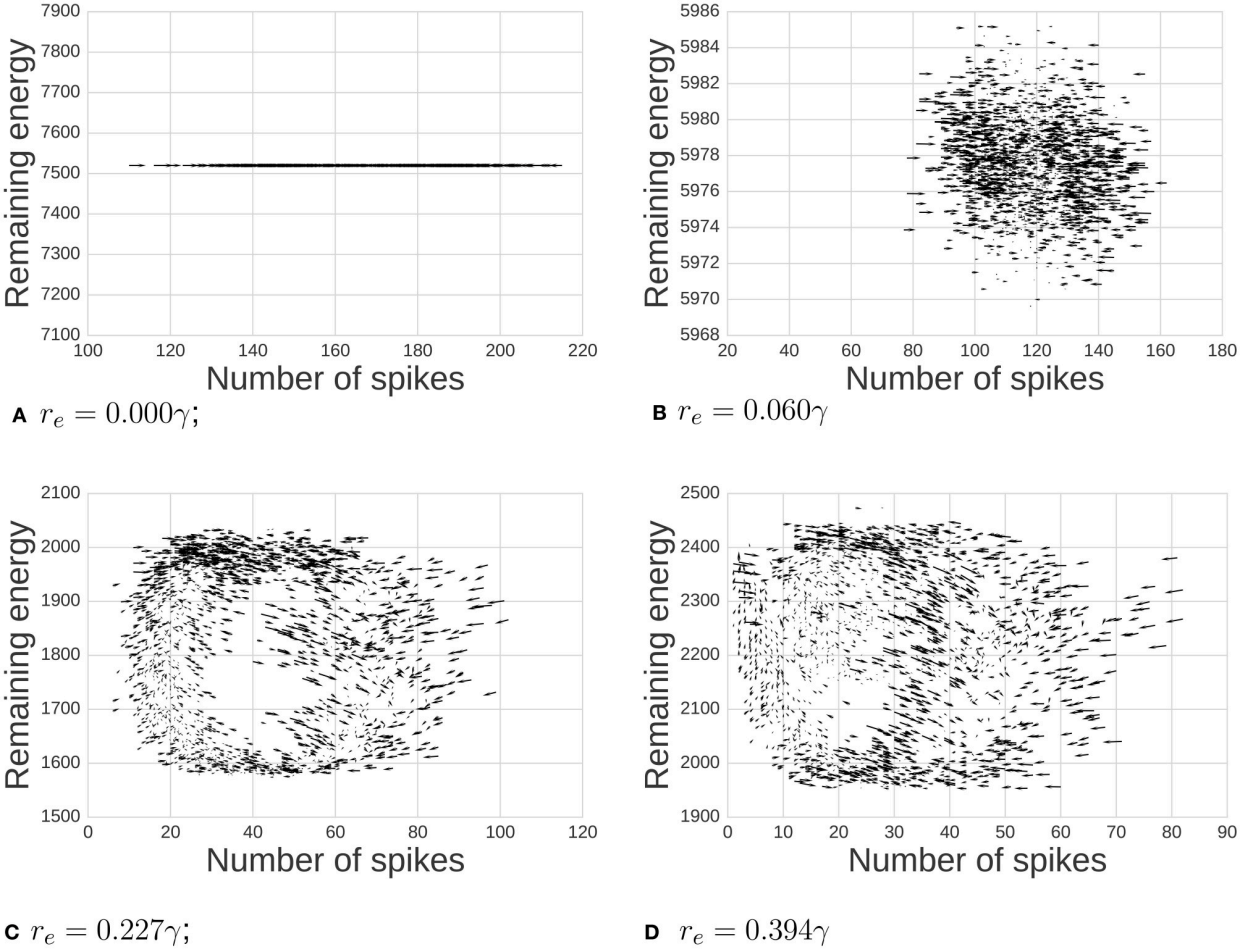
**Dynamics between available energy and total activity of the network**. Arrows shows how the system will likely move at each point (the gradient of the system). As we increase the energy requirement *r*_*e*_, we move from a state of noisy independent points to a more classic limit cycle. We also observe notable cases: **(A)** when *r*_*e*_ = 0γ there is no consumption of energy at all, **(B,C)** middle ranges, and **(D)** when *r*_*e*_ is high enough, the system reaches the 0 activity level thus breaking the cycle's dynamics.

Our chief finding is emergent network oscillations, which arise when energy demands are present, and may be reminiscent of oscillations found in the brain. Energy requirements regulate levels of activity in this network of spiking neurons (Figure 2). For some parameter ranges, the system itself is a self-sustained oscillator, notably even when energy regeneration rates are constant. To understand the relation of this model with common self-sustained oscillators, we can analyze it as an instance of the class of Lotka-Volterra (LV) predator-prey models. The LV equations often describe dynamics of two species interacting in an ecosystem. To survive and reproduce, the predator consumes the prey. When the number of prey increases, the predators have more available food, increasing their population by reproducing, which in turn reduces the prey population, continuing full-circle. This dynamic can be seen in a phase space plot or limit cycle (Figure 3).

We can use the metaphor provided by the LV model to understand our neuronal population with energy requirements. In this case, the available energy in the pool acts as the prey, required for the neuron to spike. The neuronal spikes act analogously to predators, requiring energy in order to generate, and diminishing energy pools with a stochastic “reproduction rate” given by the probability of producing downstream spikes. There are several interesting features of our neuronal ecosystem. First, the predator, a spike, is not deterministic, as spikes may be born spontaneously as a consequence of the neuron model. Second, for a new predator to be created there should be available energy in the associated local energy pool of that particular neuron. Recall that in the LV model, the overall number of prey is the value that counts, while in true evolutionary systems and our neuron model, locality matters. Here, there could be zero activity even when the number of available pools is non-zero (but small), though that context also exists in classic evolutionary applications of LV. Third, if we exclude spontaneous generation, reproduction only happens between connected elements, creating network effects, common in spiking neural networks.

### 3.2. Oscillatory dynamics emerge as a limit-cycle

First, we demonstrate the influence of *energy per spike* on emergent oscillations. The phase space plot of our model is depicted in Figure 3. Arrows show a tendency toward statistical directionality in the system. When there is no energy requirement (*r*_*e*_ = 0γ), the level of energy is constant and the amount of activity varies independently of energy. With *r*_*e*_ small (0.06γ), there are variations in level of activity, but dynamics are not primarily related to position in phase space. As we increase energy requirements (*r*_*e*_ = 0.277γ), the system organizes itself and a limit cycle emerges. Finally, when *r*_*e*_ = 0.394γ, the system rhythmically dies due to a lack of available energy in pools supporting neurons.

### 3.3. Cost per spike influences synchrony

**Figure 4:**
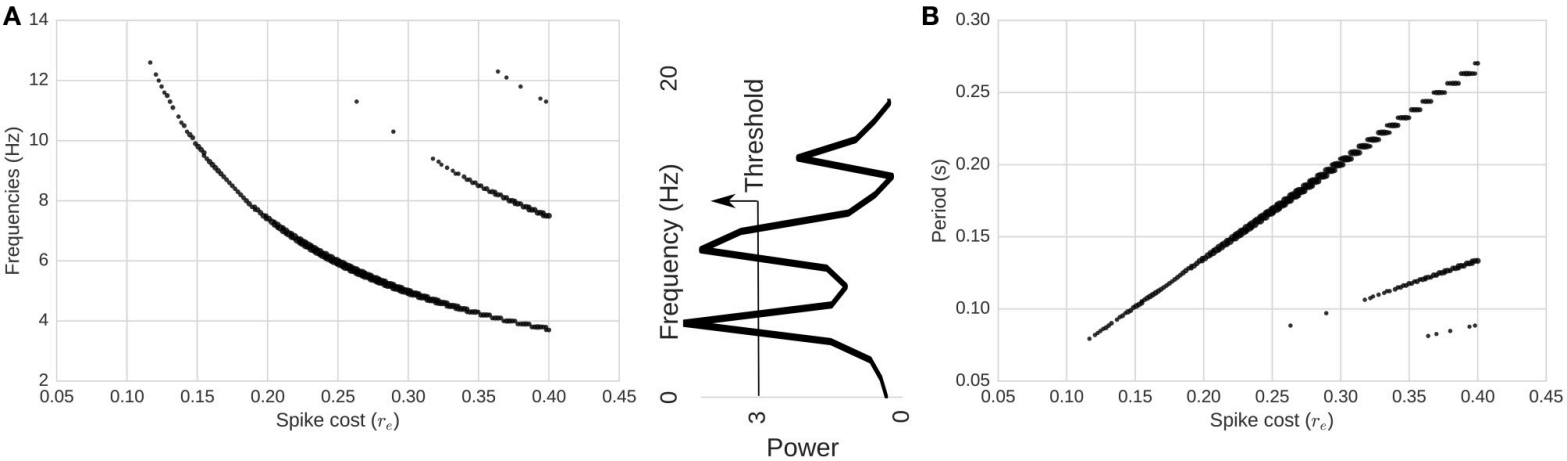
**Relationship between spike-cost (***r***_***e***_; x-axis) and oscillatory frequency distribution (y-axis). (A)** Frequency and **(B)** period of the components above a power threshold of three as a function of *r*_*e*_. Middle image depicts power threshold process which isolates the signal of peaks. As we increase the required energy, the frequency of the total activity oscillation decreases, while period increases. Changes in *r*_*e*_ produce linear changes in the period of the oscillations. Secondary smaller parallel curves correspond to the *extinction* of spiking activity due to too few resources.

Next, we compute the power spectrum of network activity (Figures 2, 4) as a means to better understand periodic behavior in the network. As required energy per spike, *r*_*e*_, increases, some frequencies stand out. There is a relationship between the dominant frequencies and the magnitude of *r*_*e*_. To show this visually, in Figure 4 we plot, for each *r*_*e*_, frequencies of the spectrum above a fixed power threshold, i.e., above 3, which arbitrarily isolates the signal of the power peaks in this case. These plots show that the frequency of the largest periodic component decreases as we increase the energy requirement per spike. When we plot the same data illustrating period instead of frequency, the relation turns out to be linear (Figure 4). As we increase the spike cost requirement, the period of the wave increases (the time between two peaks in activity increases). The slope of the larger line is related to the replenishment rate τ_*e*_. Using τ_*e*_ = 0.003γ/*ms*, the slope that relates energy to period is 0.6814*ms*/γ (*STD* = 0.001). If we double the replenishment rate to 0.006γ/*ms*, the slope becomes 0.3347*ms*/γ (*STD* = 0.001), which is almost half the original slope (not depicted graphically). Behavior remains similar, but it takes half the time to go from one peak in activity to the next, as the energy going into the system in one time-step is doubled.

### 3.4. Harmonics emerge near energetic limits

Interestingly, at high energy requirements we find secondary components (harmonics) with different slopes. Those harmonics are a consequence of the limit cycle passing through a zero-activity level. In the LV context, this is equivalent to an extinction of the predator population (spiking activity). This is shown in Figure 3D. As a consequence, we see a range in the spectrum of energy requirements between 0γ and approximately 0.26γ in our setup (with no harmonics), where the period of oscillations can be adjusted linearly while having a stable system. These appear more likely to be biological proportions, as elaborated in the Discussion below.

### 3.5. Energetic cost demonstrates a complex relationship with activity

**Figure 5:**
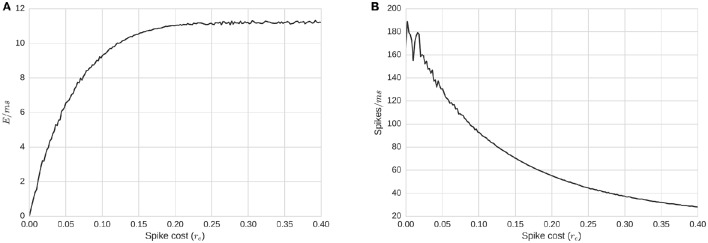
**Energy consumption and firing rate of the whole network as a function of ***r***_***e***_, required energy for a single spike. (A)** Average energy consumption rate for the whole network per time-step, as a function of *r*_*e*_. **(B)** Average total activity of the whole network as a function of *r*_*e*_. Plots show that average energy consumption rate is a concave function of *r*_*e*_. This implies that *r*_*e*_ actually affects the level of activity in a non-linear manner. Biological ranges are likely around the concave minima of about *r*_*e*_ = 0.15.

Relationships between energy requirement per spike, *r*_*e*_, and the level of *total activity* can be seen in Figure 5. With a complete simulation of T time-steps, summing all the energy consumption over the simulation and dividing by T produces the average energy consumption rate for the whole network per time-step, $E/ms=\frac{\overset{T}{\underset{0}{\sum }}E_{t}}{T}$ (Figure 5). The same average energy consumption per millisecond, alternatively defined via translation from spike/activity counts, $E/ms=Ē=\bar{\text{activity}}\left(r_{e}\right)\;\times r_{e}$, is a non-decreasing concave function of *r*_*e*_ (Figure 5). As we increase *r*_*e*_, Ē increases but at a slower rate, implying that the activity level decreases as a function of *r*_*e*_ (Figure 5). If the relation between the level of activity and the required energy is null, the average energy consumption per millisecond would be a straight line: Ē = *activity* × *r*_*e*_. The level of activity is negatively affected by *r*_*e*_; thus, the slope of Ē(*r*_*e*_) decreases. For *r*_*e*_ large enough, the function approaches an asymptote. To understand the meaning of this asymptote, we can hypothesize an energy level *r*_*e*_ and activity level, *A*, which changes at the next step to $r_{e}^{\prime }$, *A*′ where $r_{e}^{\prime }=2r_{e}$. For the average energy consumption per ms to remain the same, it is required that:

(4) $$\begin{array}{l} r_{e}A=r_{e}^{\prime }A^{\prime }=2r_{e}A^{\prime }. \end{array}$$

If we consider the network as a binomial model with the expected activity as *A* = *p*(*r*_*e*_)*N*, where *p*(*r*_*e*_) is the probability for a neuron firing for a given *r*_*e*_, then for Equation (4) to hold, it is required that $p\left(r_{e}^{\prime }\right)=\frac{1}{2}p\left(r_{e}\right)$ or $2p\left(r_{e}^{\prime }\right)=p\left(r_{e}\right)$. This model assumes independence of firing. We need twice as much energy to generate the same amount of activity. Given that the energy replenishment is fixed, the system needs twice as much time to generate and use the energy required for *A*, the given level of activity.

**Figure 6:**
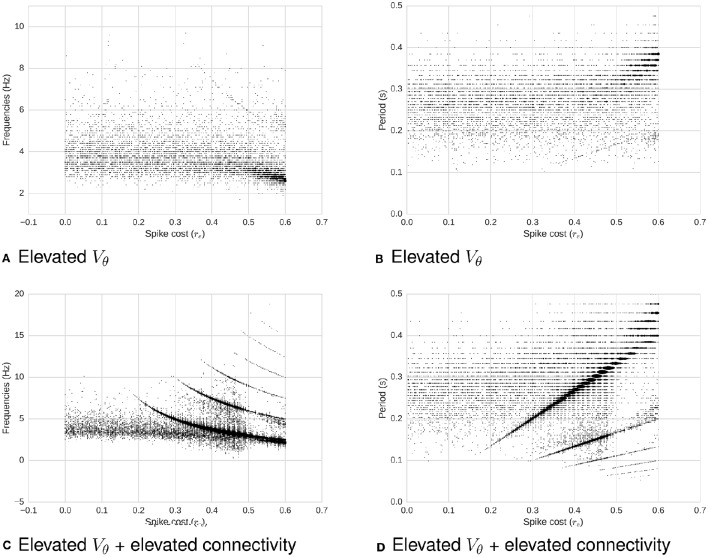
**Frequency (left) and period (right) as a function of required energy ***r***_***e***_, with increased membrane potential threshold ***V***_**θ**_ (all) and increased connectivity (bottom). (A)** Frequency as a function of *r*_*e*_ for high *V*_θ_. **(B)** Period as a function of *r*_*e*_ for high *V*_θ_. **(C)** Frequency as a function of *r*_*e*_ for high *V*_θ_ and high connectivity. **(D)** Period as a function of *r*_*e*_ for high *V*_θ_ and high connectivity. When *V*_θ_ increases with a low connectivity (top two plots), membrane potential dominate the system's dynamics: There is no clear relation between *r*_*e*_ and oscillations. On the other hand, when the connectivity increases (bottom two plots), making each neuron more likely to fire due to neighbors' input spikes, a relation between *r*_*e*_ and periodicity of oscillations re-emerges. To illustrate this relationship, the power threshold for the spectrum can be decreased, which in turns causes the appearance of noise.

The asymptote that appears in Figure 5 shows the existence of a notable situation: when the energy's dynamics dominate the activity dynamics (around fire energy > 0.15). In many spike-response or integrate and fire models, a neuron will fire even with no incoming signals, albeit at a minimum frequency. In our model, membrane potential has a fixed decay toward the firing threshold, and the minimum firing frequency without inputs is controlled by the relation between the resting state *V*_*r*_ voltage, threshold voltage *V*_θ_, and the decay τ_*v*_. If the frequency of firing is faster than the time required for a pool to regenerate sufficiently for another spike after firing, energy would be the primary limiting factor for firing, rather than input spikes (via membrane potential); this would be energy dominating the system. On the other hand, if the energy replenishes faster than the average time required for inputs to cause the membrane potential to reach a threshold, energy will play little role in the dynamics. Finally, there are situations in between, when neither the energy nor the membrane potential completely dominates the dynamics. This middle range is of most interest. To further observe these dynamics, we replicate simulations in two further experiments:

1. Increasing the membrane potential threshold *V*_θ_ (Figure 6).
2. Also keeping a high *V*_θ_, and additionally increasing the connectivity of the network while preserving the proportional correlation matrix (Figure 6).

This demonstrates a second continuum, revealing the varying influence of energetic costs on the system's behavior. We now discuss the implications of these results.

## 4. Discussion and conclusions

Energy, time, and many neuronal parameters in our study are, in actuality, unit-less, and like any symbolic model, the proportions of these parameters, rather than their absolute values, define accuracy. The goal of our approach is to systematically map the space of energy and neuronal parameters, not only to cover biological ranges, but to extend parameters widely enough to describe the effect of energy on networked systems generally. In biological systems, neither the action potentials alone nor energy alone dominate neuronal firing, with both explaining variation in experimental evidence. Our experiments include a wide range of combined proportional parameters, within which likely exist biologically realistic proportions for corresponding features. Overall, some ranges appeared biologically realistic and some limit-ranges did not. For example, with no required energy the network will typically remain highly active for long periods of time, while with higher energy requirements for a spike, activity can sometimes dampen or die out, with a continuum of impact in between these ranges. In middle ranges, the system demonstrates more realistic sustained and lively bursting.

### 4.1. Input parameter matching

The multiple energy parameters of the network are initialized at varying proportions, and it is an open question which ranges of ratios are realistic. Several proportions may be considered:

1. The ratio of *spike cost* to *total pool quantity* determines the number of spikes required to empty the pool, in the absence of any regeneration of the pool. Studies of ATP imaging and synthesis blockage at the synapse suggest this ratio should result in a fairly rapid time to empty the immediately available energy under constant spiking conditions with no hypothetical energy regeneration (Rangaraju et al., 2014).
2. The ratio of *spike cost* to *regeneration rate* (defined both by frequency and magnitude) determines which ranges of spike rate form an equilibrium with the regeneration rate. Realistic ranges may be obtained by (a) interpolating limits to avoid extinction or saturation of random firing, (b) determining which produce a realistic firing rate, (c) determining which produce realistic firing rate distributions and limit-cycle behavior, discussed in the next subsection.
3. The ratio of *regeneration rate* (defined both by frequency and magnitude) to *total pool quantity* can potentially define the depth or type of oscillations that emerge, due to the possibility of harmonic interactions between neuronal oscillations and regeneration caps. Mapping the space of these interactions would be an extensive undertaking, and so we defined this ratio at only two levels, with both parameters at a high frequency to avoid any such interactions.

### 4.2. Emergent output matching and interpretation

With varying levels of spike cost in relation to other parameters, we can ask which outputs might be most realistic, and at such hypothesized proportions, whether network behavior produces any notable emergent features:

1. Firing rate (frequency) distribution represented on the right-most column of Figure 2 demonstrates a realistic frequency distribution as often observed in EEG. Specifically, EEG data have a fat-tailed geometric distribution with large quantities of low frequencies (0−5 Hz), tapering off rapidly just below alpha (10 Hz), and continuing to taper much more slowly past 20 Hz. In our results, the range between *r*_*e*_ = 0.006 and *r*_*e*_ = 0.227γ produces a distribution which appears to match such EEG power spectra.
2. Another picture of the frequency distributions can be obtained from the limit cycle plots (Figure 3). Too high levels of energy requirements produce extinction, with too low producing random saturated firing. A value between *r*_*e*_ = 0.006 and *r*_*e*_ = 0.227γ produces moderate behavior, which we hypothesize to be realistic.
3. To improve upon the previous two snapshots, the energy consumption and firing rates as a function of spike cost may also inform realistic ranges with a more continuous resolution (Figure 5). The convex maxim for energy consumption around *r*_*e*_ = 0.15γ serves as the mid-point between firing and energy dominating activity. The same point serves as a stable minima in the concave function describing quantity of firing, perhaps acting as an attractor, producing an efficient means to generate low levels of self-sustained activity, which is itself often considered difficult to cultivate experimentally in neural networks (Gewaltig, 2013).
4. To extend the previous point, realistic total quantities of firing can be observed at this maxima/minima of *r*_*e*_ = 0.15γ. Published extrapolations suggest that the cost of a spike is so high that <1% of neurons can be “substantially active concurrently” (Lennie, 2003). Indeed, in the time window we bin into, the range of percentage active at a single point in time is just slightly below the 1% range.

### 4.3. Level of abstraction

We do not take into consideration costs of non-signaling activity (Hyder et al., 2013b), variable requirements of gray vs. white matter (Harris and Attwell, 2012), or dynamics of particular metabolites in neuron-glia energetics (Jolivet et al., 2009, 2010, 2015). Instead we focus on network dynamics at a higher level of abstraction, attempting to create realistic global behavior, which could serve as a means of time-series estimation.

### 4.4. Functional neuroenergetics via non-invasive imaging

Using MR techniques to measure oxygen consumption can serve as a good metabolic marker for exploring brain metabolism directly (Hyder et al., 2011; Shu et al., 2016). Measuring specific metabolites such as glucose or ketone bodies using PET is also possible (Wang et al., 2003; Bentourkia et al., 2009; Pifferi et al., 2011; Di and Biswal, 2012). Using such methods, studies have shown that loss of consciousness due to anesthesia largely reduces energy consumption, and that the awake resting brain at baseline requires a large proportion of the brain's energy budget (Shulman et al., 2004, 2009; Hyder et al., 2013a). Though EEG data have no reliable spatial resolution, they can serve as an indirect means to asses gross metabolic changes (Hyder et al., 2013b). For example, glucose consumption after fasting has a detectable impact on resting state EEG (An et al., 2015); indeed, in our model we see increases in similar frequency bands with mid-range energy requirements.

### 4.5. Cost of neuronal energetics within and across species

We employed connectivity data from human subjects, and there is some question as to whether the greater neuronal density in primates vs. rodents may influence energetic requirements. It appears that energetic cost may scale linearly with the number of neurons in both rodents and primates (Herculano-Houzel, 2011b; Balaban, 2013; Hyder et al., 2013b). The resting state methods used to infer one of the networks we used have also been employed in studying energetics. Resting state functional connectivity may indeed be metabolically expensive, albeit with hubs demonstrating possibly higher energy efficiency (Tomasi et al., 2013). Local glucose consumption may influence the degree of resting state functional connectivity (Riedl et al., 2014). Metabolic networks measured via FDG-PET can be analyzed much like rsfMRI data would be, producing covariant metabolic networks which largely overlap with rs-fMRI networks (Di and Biswal, 2012; Thompson et al., 2016). Further, impeding mitochondrial function can impact resting state functional connectivity (Sanganahalli et al., 2013). Neural networks have been employed to illustrate some of the dynamics between active and resting state activity (Bennett et al., 2015), and for integrating EEG, fMRI, and metabolic demands (Sotero and Trujillo-Barreto, 2008). Overall, it is likely the case that at our general level of abstraction, that our energetic model may complement such studies both in animals and humans.

### 4.6. Biological speculation

Biologically, energy may not just be used supply basic metabolic cellular needs, but energy itself may be a domain of information processing much like membrane potential and spiking, with ATP acting as a major excitatory neurotransmitter, and adenosine acting as a major inhibitory neurotransmitter (Lindberg et al., 2015). This may not just be cross-talk between independent systems, but may in part explain the algorithmic role glia play in signaling. Glia do indeed likely play a role in modulating neuronal signaling, perhaps performing slower time-scale associative learning (Barres, 2008; Araque and Navarrete, 2010), complementing the faster time-scale learning via spike-timing dependent plasticity (STDP) (Taylor, 1973; Levy and Steward, 1983; Dan and Poo, 1992; Debanne et al., 1994; Markram et al., 1997; Bi and Poo, 1998). Neurons may integrate many different types of signals, including cross-talk between spikes, homeostatic events, energy, and others for the purposes of information processing, learning, and pattern detection. Our model is congruent with the feasibility of such interactions, with a complex interplay between energetic oscillations and neuronal activity.

### 4.7. Technological speculation

Our results may extend beyond informing studies of brain and biology. As introduced above with computerized load balancing and sharing, network connectivity maintenance, predator-prey networks, and economics (Lotka, 1910; Goel et al., 1971; Gandolfo, 2008; Wan et al., 2008; Allan et al., 2010; Shtessel et al., 2012; Arpaci-Dusseau and Arpaci-Dusseau, 2015), we assert that resource-utilization generally, causes the emergence of oscillations and periodic components. Here, we illustrate an analogous relationship, with oscillatory dynamics and energy requirements in the brain. We propose that such dynamics necessarily must emerge with two features of (1) a momentum-like delay and (2) bi-directional interdependence. Each of the above and our neural networks should be considered special cases only. Thus, our model has implication beyond critical inclusion in computational neuroscience and may be of relevance to future energy-aware technological networks.

## Author contributions

JB programmed and designed data analysis with guidance from PT and HS. PT designed initial model and experiments with input from HS, CC, and TV. Together, CC, TV, and JB programmed model framework with guidance from PT and HS. PT, JB, and HS wrote manuscript. HS supervised all research.

### Conflict of interest statement

The authors declare that the research was conducted in the absence of any commercial or financial relationships that could be construed as a potential conflict of interest. The reviewer DR and handling Editor declared their shared affiliation, and the handling Editor states that the process nevertheless met the standards of a fair and objective review.
